# Vildagliptin Has a Neutral Association With Dementia Risk in Type 2 Diabetes Patients

**DOI:** 10.3389/fendo.2021.637392

**Published:** 2021-04-30

**Authors:** Chin-Hsiao Tseng

**Affiliations:** ^1^ Department of Internal Medicine, National Taiwan University College of Medicine, Taipei, Taiwan; ^2^ Division of Endocrinology and Metabolism, Department of Internal Medicine, National Taiwan University Hospital, Taipei, Taiwan; ^3^ Division of Environmental Health and Occupational Medicine of the National Health Research Institutes, Zhunan, Taiwan

**Keywords:** vildagliptin, dementia, diabetes mellitus, pharmacoepidemiology, Taiwan

## Abstract

**Background and aims:**

Animal studies suggested that vildagliptin might exert a beneficial effect on cognitive function. The present study evaluated whether the use of vildagliptin in patients with type 2 diabetes mellitus might affect dementia risk.

**Methods:**

The database of Taiwan’s National Health Insurance was used to enroll an unmatched cohort and a propensity score-matched-pair cohort of ever and never users of vildagliptin from patients with newly diagnosed diabetes mellitus during 2002-2014. The patients should be alive on January 1, 2015 and were followed up for dementia diagnosis until December 31, 2016. Unadjusted and multivariate-adjusted hazard ratios (HR) and their 95% confidence intervals (CI) were estimated for vildagliptin ever *versus* never users, for cumulative duration and cumulative dose of vildagliptin therapy categorized into tertiles *versus* never users, and for cumulative duration and cumulative dose treated as continuous variables.

**Results:**

There were 355610 never users and 43196 ever users in the unmatched cohort and 40489 never users and 40489 ever users in the matched cohort. In the unmatched cohort, unadjusted HR (95% CI) was 0.929 (0.683-1.264) and the multivariate-adjusted HR (95% CI) was 0.922 (0.620-1.372). In the matched cohort, the unadjusted HR (95% CI) was 0.930 (0.616-1.402) and the multivariate-adjusted HR (95% CI) was 0.825 (0.498-1.367). None of the analyses conducted for cumulative duration and cumulative dose was significant, either being treated as tertile cutoffs or as continuous variables, in either the unmatched cohort or the matched cohort.

**Conclusions:**

This study showed a neutral effect of vildagliptin on dementia risk.

## Introduction

Both diabetes and dementia affect hundreds of millions of the world population. The International Diabetes Federation estimates that 463 million people or 1 in every 11 adults aged 20 to 79 years have diabetes mellitus over the world (1). On the other hand, the World Health Organization estimates that around 50 million people are suffering from dementia over the world and every year there are nearly 10 million new cases of dementia (2). Diabetes mellitus and dementia are closely linked and diabetes patients may have a significantly higher risk of dementia. According to a meta-analysis that included 20 studies, diabetes mellitus is associated with an approximately 70% higher risk of all types of dementia (3). Studies conducted in Taiwan using the reimbursement database of the National Health Insurance (NHI) showed a similarly increased risk of dementia of 50% (4) to 60% (5) in the diabetes patients. Dementia can be resulted from either a vascular etiology or a neurodegenerative disease known as Alzheimer’s disease (which contributes to 60-70% of the cases of dementia) (2). The two disease entities may share common pathophysiological changes of impaired insulin expression and insulin resistance, leading to the coining of “type 3 diabetes” for Alzheimer’s disease (6). The higher risk of dementia in diabetes patients may also be explained by vascular and metabolic changes associated with hyperglycemia and diabetes-related comorbidities, including atherosclerosis, increased deposition of advanced glycation end-products, dysregulation of lipid metabolism, and augmented status of inflammation and oxidative stress (6, 7).

Dipeptidyl peptidase-4 (DPP4) inhibitors are commonly used oral antidiabetic drugs that stimulate insulin secretion by prolonging the half-life of glucagon like peptide-1 and glucose dependent insulinotropic polypeptide (8). Vildagliptin is one of the drugs in the class of DPP4 inhibitors. Previous *in vitro* and animal studies suggested that vildagliptin might improve cognitive dysfunction, exert neuroprotective effect and prevent the development of Alzheimer’s disease or dementia (9–17). A recent animal study from China suggested that vildagliptin might alleviate cognitive deficits of spatial learning and memory by using the Morris water maze in streptozotocin-induced diabetes in male Wistar rats (18). Such a beneficial effect might be exerted through reducing the levels of apoptosis-related proteins in the hippocampus, probably *via* reversing diabetes-induced decrease in the phosphorylated (p)-protein kinase B (Akt) and p-glycogen synthase kinase 3β (18). However, whether this neuroprotective effect observed in *in vitro* and animal studies could be applied to millions of patients with type 2 diabetes mellitus who had been treated with vildagliptin has not been answered. The purpose of the present study was to compare the dementia risk in patients with type 2 diabetes mellitus who had been treated with vildagliptin to those who had never been treated with vildagliptin by using the reimbursement database of the Taiwan’s NHI.

## Materials and Methods

This is a retrospective cohort study that used the longitudinal reimbursement database of Taiwan’s NHI, which has been implemented since March 1995. The NHI is a unique and compulsive healthcare system covering >99.9% of Taiwan’s population. The Bureau of NHI signed contracts with all in-hospitals and 93% of all medical settings throughout Taiwan.

The database is managed by the Ministry of Health and Welfare and keeps all records of disease diagnoses, medication prescriptions and performed procedures. It can be used for academic research after ethics review and the study was approved by the Research Ethics Committee C of the National Taiwan University Hospital (NTUH-REC No. 201805002WC). On-site analyses were conducted at the Health and Welfare Data Center of the Ministry. Informed consent was not required according to local regulations because the database had been de-identified before release for analyses for the protection of privacy.

Throughout the study period, diabetes mellitus was coded 250.XX according to the International Classification of Diseases, Ninth Revision, Clinical Modification (ICD-9-CM) and dementia was coded as abridged codes of A210 or A222, or as ICD-9-CM codes of 290.0, 290.1, 290.2, 290.4, 294.1, 331.0–331.2, or 331.7–331.9.

**Figure 1:**
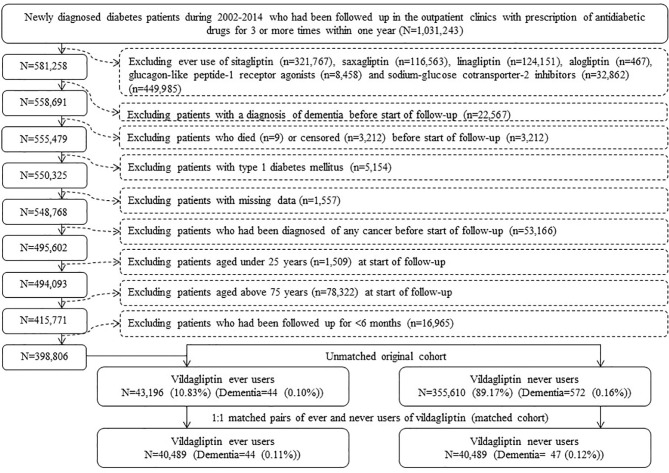
Flowchart showing the procedures followed in creating the unmatched original cohort and a cohort of 1:1 matched pairs of ever users and never users of vildagliptin from the reimbursement database of the National Health Insurance.

The procedures used to create an unmatched cohort and a cohort of 1:1 matched pairs of ever and never users of vildagliptin are shown in **Figure 1**. At first, 1,031,243 patients who had newly diagnosed diabetes mellitus during 2002-2014 and had been prescribed antidiabetic drugs for 3 or more times within one year were identified from the outpatient clinics. Patients who had a diagnosis of diabetes mellitus in 2001 or before were excluded to ensure a new diagnosis after 2002. The following patients were then excluded: 1) 449,985 patients who had ever used other incretin-based therapies [including sitagliptin (n = 321,767), saxagliptin (n = 116,563), linagliptin (n = 124,151), alogliptin (n=467) and glucagon like peptide-1 receptor agonists (n = 8,458)] and/or sodium-glucose cotransporter-2 inhibitors (n = 32,862); 2) 22,567 patients with a diagnosis of dementia before start of follow-up; 3) 3,212 patients who died (n = 9) or censored ( n= 3,212) before start of follow-up; 4) 5,154 patients with type 1 diabetes mellitus; 5) 1,557 patients with missing data; 6) 53,166 patients who had been diagnosed of any cancer before start of follow-up (cancer patients were excluded because they might have shortened lifespan and might have distorted follow-up time, and dementia could be misdiagnosed from the clinical presentations of malignancy); 7) 1,509 patients aged <25 years at start of follow-up; 8) 78,322 patients aged >75 years at start of follow-up [the life expectancy of the Taiwan population at the beginning of the study in the year 2000 was approximately 75 years (19), therefore inclusion of older patients might tend to suffer from “healthy survivor” bias (20)]; and 9) 16,965 patients with a follow-up duration <6 months. As a result, 43,196 ever users and 355,610 never users of vildagliptin were identified (unmatched original cohort). A cohort of 1:1 matched pairs of 40,489 ever users and 40,489 never users (the matched cohort) was created by matching on propensity score based on the Greedy 8→1 digit match algorithm (21). Logistic regression was used to create the propensity score from all characteristics listed in **Table 1**.

**Table 1:** Characteristics of never users and ever users of vildagliptin in the unmatched original cohort and in the matched cohort.

Variable	Unmatched cohort					Matched cohort				
	Never users		Ever users		*P* value	Never users		Ever users		*P* value
	(*n *= 355610)		(*n *= 43196)			(*n *= 40489)		(*n *= 40489)		
	*n*	%	*n*	%		*n*	%	*n*	%	
**Basic data**										
Age (years)	59.10	9.19	58.19	9.37	<0.0001	58.43	9.63	58.16	9.36	<0.0001
Diabetes duration (years)	5.26	3.47	6.02	3.61	<0.0001	5.59	3.49	6.00	3.62	<0.0001
Sex (men)	160756	45.21	18769	43.45	<0.0001	17854	44.10	17616	43.51	0.0918
Occupation										
I	149742	42.11	18650	43.18	<0.0001	16854	41.63	17515	43.26	<0.0001
II	73458	20.66	9027	20.90		8419	20.79	8499	20.99	
III	62847	17.67	7333	16.98		6962	17.19	6831	16.87	
IV	69563	19.56	8186	18.95		8254	20.39	7644	18.88	
Living region										
Taipei	114624	32.23	13080	30.28	<0.0001	13146	32.47	12453	30.76	<0.0001
Northern	47693	13.41	5200	12.04		5480	13.53	4876	12.04	
Central	63151	17.76	11437	26.48		7286	18.00	10611	26.21	
Southern	57087	16.05	5798	13.42		6364	15.72	5369	13.26	
Kao-Ping and Eastern	73055	20.54	7681	17.78		8213	20.28	7180	17.73	
**Major comorbidities**										
Hypertension	186307	52.39	23212	53.74	<0.0001	21474	53.04	21383	52.81	0.5217
Dyslipidemia	177612	49.95	22271	51.56	<0.0001	20558	50.77	20537	50.72	0.8827
Obesity	10325	2.90	1407	3.26	<0.0001	1215	3.00	1363	3.37	0.0031
**Diabetes-related complications**										
Nephropathy	62464	17.57	8271	19.15	<0.0001	7562	18.68	7735	19.10	0.1204
Eye disease	53665	15.09	7119	16.48	<0.0001	6458	15.95	6684	16.51	0.0312
Stroke	65665	18.47	8569	19.84	<0.0001	7895	19.50	8018	19.80	0.2767
Ischemic heart disease	87513	24.61	11286	26.13	<0.0001	10394	25.67	10466	25.85	0.5629
Peripheral arterial disease	45352	12.75	6038	13.98	<0.0001	5486	13.55	5690	14.05	0.0377
**Major risk factors of dementia**										
Head injury	13719	3.86	1898	4.39	<0.0001	1663	4.11	1800	4.45	0.0173
Parkinson’s disease	11482	3.23	1574	3.64	<0.0001	1354	3.34	1511	3.73	0.0028
Hypoglycemia	40255	11.32	5389	12.48	<0.0001	4729	11.68	5090	12.57	0.0001
Encephalitis and/or meningoencephalitis	657	0.18	122	0.28	<0.0001	76	0.19	122	0.30	0.0011
Osteoporosis	32089	9.02	4358	10.09	<0.0001	3837	9.48	4115	10.16	0.0010
Muscular wasting	3240	0.91	465	1.08	0.0007	402	0.99	465	1.15	0.0315
Accidental falls	1088	0.31	176	0.41	0.0004	126	0.31	176	0.43	0.0039
**Potential risk factors of cancer**										
Chronic obstructive pulmonary disease	91784	25.81	11881	27.50	<0.0001	10928	26.99	10982	27.12	0.6693
Tobacco abuse	10173	2.86	1423	3.29	<0.0001	1223	3.02	1369	3.38	0.0036
Alcohol-related diagnoses	12699	3.57	1703	3.94	<0.0001	1496	3.69	1634	4.04	0.0119
Gallstone	23425	6.59	3213	7.44	<0.0001	2802	6.92	3001	7.41	0.0067
Diseases of the digestive system	226288	63.63	28000	64.82	<0.0001	26058	64.36	25675	63.41	0.0051
Hepatitis B virus infection	15351	4.32	2129	4.93	<0.0001	1808	4.47	1808	4.47	0.0005
Hepatitis C virus infection	14779	4.16	1983	4.59	<0.0001	1730	4.27	1885	4.66	0.0084
Liver cirrhosis	14651	4.12	1984	4.59	<0.0001	1733	4.28	1885	4.66	0.0097
**Antidiabetic drugs**										
Sulfonylurea	85333	24.00	10738	24.86	<0.0001	10258	25.34	10519	25.98	0.0357
Metformin	10738	3.02	14829	34.33	<0.0001	16013	39.55	14829	36.62	<0.0001
Meglitinide	8068	2.27	1182	2.74	<0.0001	2490	6.15	1031	2.55	<0.0001
Acarbose	12797	3.60	1754	4.06	<0.0001	4018	9.92	1527	3.77	<0.0001
Thiazolidinediones	16605	4.67	1461	3.38	<0.0001	4705	11.62	1275	3.15	<0.0001
**Medications commonly used in diabetes patients**										
Angiotensin converting enzyme inhibitor/angiotensin receptor blocker	207170	58.26	28050	64.94	<0.0001	23458	57.94	26051	64.34	<0.0001
Calcium channel blocker	186126	52.34	22377	51.80	0.0350	21172	52.29	20885	51.58	0.0435
Statin	230140	64.72	30442	70.47	<0.0001	26078	64.41	28487	70.36	<0.0001
Fibrate	99162	27.89	13790	31.92	<0.0001	11624	28.71	11624	28.71	<0.0001
Aspirin	117604	33.07	16909	39.14	<0.0001	13520	33.39	15715	38.81	<0.0001

Age and diabetes duration are expressed as mean and standard deviation.

Cumulative duration (in months) and cumulative dose (in mg) of vildagliptin therapy were calculated from the database. Potential confounders included the following categories: basic data, major comorbidities associated with diabetes mellitus, diabetes-related complications, major risk factors of dementia, potential risk factors of cancer, antidiabetic drugs and medications commonly used in diabetes patients. Basic data included age, diabetes duration, sex, occupation and living region (classified as Taipei, Northern, Central, Southern, and Kao-Ping/Eastern). Occupation was classified as class I (civil servants, teachers, employees of governmental or private businesses, professionals and technicians), class II (people without a specific employer, self-employed people or seamen), class III (farmers or fishermen) and class IV (low-income families supported by social welfare, or veterans). Major comorbidities associated with diabetes mellitus included hypertension (ICD-9-CM 401-405), dyslipidemia (272.0-272.4) and obesity (278). Major diabetes-related complications included nephropathy (580-589), eye diseases (250.5: diabetes with ophthalmic manifestations, 362.0: diabetic retinopathy, 369: blindness and low vision, 366.41: diabetic cataract, and 365.44: glaucoma associated with systemic syndromes), stroke (430-438), ischemic heart disease (410-414) and peripheral arterial disease (250.7, 785.4, 443.81 and 440-448). Potential risk factors of dementia included head injury (959.01), Parkinson’s disease (332), hypoglycemia (251.0, 251.1 and 251.2), encephalitis and/or meningoencephalitis (323, 062, 063, 064 and 054.3), osteoporosis (733.00), muscular wasting (728.2) and accidental falls (E880-E888). Potential risk factors of cancer included chronic obstructive pulmonary disease (a surrogate for smoking, 490-496), tobacco abuse (305.1, 649.0 and 989.84), alcohol-related diagnoses (291, 303, 535.3, 571.0-571.3 and 980.0), gallstone (574.00, 574.01, 574.10, 574.11, 574.20, 574.21 and A348), diseases of the digestive system (520-579), hepatitis B virus infection (070.22, 070.23, 070.32, 070.33 and V02.61), hepatitis C virus infection (070.41, 070.44, 070.51, 070.54 and V02.62), and liver cirrhosis (571.5). Antidiabetic drugs included sulfonylurea, metformin, meglitinide, acarbose and thiazolidinediones. Commonly used medications in diabetes patients included angiotensin converting enzyme inhibitor/angiotensin receptor blocker, calcium channel blocker, statin, fibrate and aspirin.

Student’s t test compared the difference of age and diabetes duration between never and ever users and Chi-square test was used for other variables.

Incidence density of dementia was calculated for never users, ever users and tertiles of cumulative duration and cumulative dose of vildagliptin therapy. The numerator was the case number of newly diagnosed dementia identified during follow-up and the denominator was the follow-up duration in person-years. Follow-up started on January 1, 2015 and ended on December 31, 2016, at the time of a new diagnosis of dementia, or on the date of death or the last reimbursement record, whichever occurred first.

Cox proportional hazards model was used to estimate the unadjusted and multivariate-adjusted hazard ratios and their 95% confidence intervals for ever users *versus* never users, for users categorized according to tertiles of cumulative duration and cumulative dose *versus* never users, and for cumulative duration (every 1-month increment) and cumulative dose (every 1-mg increment) of vildagliptin therapy being treated as continuous variables. Analyses were conducted in the unmatched cohort and the matched cohort, respectively. In the multivariate-adjusted models, all characteristics listed in **Table 1** were considered as potential confounders.

To examine whether the findings might be consistent for patients enrolled during three different periods of time, i.e., 2002-2005, 2006-2009 and 2010-2014, multivariate-adjusted models were created for the unmatched cohort enrolled during the three periods.

Analyses were conducted using SAS statistical software, version 9.4 (SAS Institute, Cary, NC). *P* < 0.05 was considered statistically significant.

## Results

**Table 1** shows the characteristics of never users and ever users of vildagliptin in the unmatched cohort and the matched cohort, respectively.

The incidence of dementia and the unadjusted and multivariate-adjusted hazard ratios by vildagliptin exposure are shown in **Table 2**. The overall hazard ratio comparing ever *versus* never users suggested a null association. Neither the cumulative duration nor the cumulative dose of vildagliptin therapy was significantly associated with the risk of dementia when these parameters were categorized into tertiles or treated as continuous variables. The findings consistently supported a null association in the unmatched cohort and the matched cohort.

**Table 2:** Incidence rates and hazard ratios of dementia by vildagliptin exposure.

Vildagliptin use	*n*	*N*	Person-year	Incidence rate	Unadjusted model			Multivariate-adjusted model		
				(per 100,000 person-years)	Hazard ratio	95% Confidence interval	*P* value	Hazard ratio	95% Confidence interval	*P* value
**Unmatched cohort**										
Vildagliptin never users	572	355610	620302.42	92.21	1.000			1.000		
Vildagliptin ever users	44	43196	64874.08	67.82	0.929	(0.683-1.264)	0.6379	0.922	(0.620-1.372)	0.6905
Tertiles of cumulative duration of vildagliptin therapy (months)										
Never users	572	355610	620302.42	92.21	1.000			1.000		
<1.87	6	15914	19428.75	30.88	0.723	(0.321-1.631)	0.4351	0.684	(0.287-1.630)	0.3918
1.87-7.47	15	12882	19812.75	75.71	0.990	(0.593-1.654)	0.9695	0.983	(0.552-1.750)	0.9545
>7.47	23	14400	25632.58	89.73	0.959	(0.632-1.455)	0.8429	0.956	(0.593-1.540)	0.8522
Cumulative duration of vildagliptin therapy treated as a continuous variable										
For every 1-month increment of vildagliptin use					0.997	(0.975-1.019)	0.7578	0.997	(0.974-1.021)	0.8299
Tertiles of cumulative dose of vildagliptin (mg)										
Never users	572	355610	620302.42	92.21	1.000			1.000		
<3,000	6	14105	17112.33	35.06	0.847	(0.375-1.911)	0.6887	0.771	(0.324-1.834)	0.5565
3,000-14,400	17	14378	21589.67	78.74	1.076	(0.663-1.744)	0.7674	1.045	(0.603-1.809)	0.8757
>14,400	21	14713	26172.08	80.24	0.858	(0.555-1.326)	0.4895	0.885	(0.540-1.450)	0.6284
Cumulative dose of vildagliptin treated as a continuous variable										
For every 1-mg increment of vildagliptin use					1.000	(1.000-1.000)	0.5452	1.000	(1.000-1.000)	0.6806
**Matched cohort**										
Vildagliptin never users	47	40489	61777.58	76.08	1.000			1.000		
Vildagliptin ever users	44	40489	62779.58	70.09	0.930	(0.616-1.402)	0.7281	0.825	(0.498-1.367)	0.4560
Tertiles of cumulative duration of vildagliptin therapy (months)										
Never users	47	40489	61777.58	76.08	1.000			1.000		
<1.87	6	13901	17876.92	33.56	0.714	(0.300-1.697)	0.4457	0.607	(0.238-1.543)	0.2939
1.87-8.37	16	12823	20339.42	78.66	0.996	(0.565-1.756)	0.9883	0.914	(0.482-1.734)	0.7839
>8.37	22	13765	24563.25	89.56	0.962	(0.578-1.599)	0.8806	0.836	(0.466-1.498)	0.5463
Cumulative duration of vildagliptin therapy treated as a continuous variable										
For every 1-month increment of vildagliptin use					0.998	(0.973-1.024)	0.8701	0.993	(0.966-1.020)	0.6131
Tertiles of cumulative dose of vildagliptin (mg)										
Never users	47	40489	61777.58	76.08	1.000			1.000		
<3,150	6	13361	17103.92	35.08	0.763	(0.321-1.816)	0.5414	0.623	(0.245-1.582)	0.3194
3,150-16,500	18	13357	21103.33	85.29	1.088	(0.632-1.872)	0.7621	0.957	(0.516-1.775)	0.8885
>16,500	20	13771	24572.33	81.39	0.872	(0.516-1.475)	0.6103	0.794	(0.436-1.446)	0.4515
Cumulative dose of vildagliptin treated as a continuous variable										
For every 1-mg increment of vildagliptin use					1.000	(1.000-1.000)	0.6062	1.000	(1.000-1.000)	0.5243

n, incident cases of dementia; N, cases followed.

**Table 3** shows the overall multivariate-adjusted hazard ratios comparing ever *versus* never users of vildagliptin analyzed in patients enrolled during three different periods, i.e., 2002-2005, 2006-2009 and 2010-2014. The results suggested a null association in all subgroups.

**Table 3:** Incidence of dementia comparing ever *versus* never users of vildagliptin in patients enrolled during three different periods of time in the unmatched cohort.

Year	Ever users		Never users		Multivariate-adjusted model		
	*n*	*N*	*n*	*N*	Hazard ratio	95% Confidence interval	*P* value
2002-2005	13	8628	125	55931	1.019	(0.529-1.963)	0.9541
2006-2009	17	13059	182	98103	1.070	(0.562-2.036)	0.8369
2010-2014	14	21509	265	201576	0.651	(0.289-1.469)	0.3016

n, incident cases of dementia; N, cases followed.

## Discussion

The findings suggested that vildagliptin use has a null association with dementia risk in patients with type 2 diabetes mellitus (**Tables 2** and **3**).

Antidiabetic drugs are being used by thousands of millions of diabetes patients. Therefore, the safety and potential pleiotropic effects or benefits of antidiabetic drugs are clinically important, especially for dementia, a disease that affects millions of patients and has a close link with diabetes mellitus. This study may have some clinical and research significance. First, neuroprotective findings of vildagliptin observed in *in vitro*, *in vivo* and animal studies should not be readily interpreted as a potential protection against dementia in humans without consideration of its accessibility to human brain. It is interesting that vildagliptin alleviated cognitive deficits of spatial learning and memory in rats with streptozotocin-induced diabetes (18), but this benefit could not be similarly demonstrated in humans in the present study (**Tables 2** and **3**). One of the possible explanations is that most DPP4 inhibitors cannot readily pass through the blood-brain barrier in humans (22). However, vildagliptin, a small molecule with a molecular weight of 303.4 Daltons (g/mol) (23), might cross the blood-brain barrier more efficiently in streptozotocin-induced diabetes rats because streptozotocin administration may cause a progressive increase in the blood-brain barrier permeability (especially significant in the midbrain) of small molecules [using vascular space markers ranging from 342 to 65,000 Daltons (g/mol) from 28 to 90 days] (24). In the animal study conducted by Zhang et al., vildagliptin was administered for 4 consecutive weeks after successful induction of diabetes by streptozotocin for 10 weeks (18). This time frame just met the time of streptozotocin-induced progressive increase of blood-brain barrier permeability observed by Huber et al. (24). Although diabetes mellitus has been claimed to affect the permeability of blood-brain barrier, findings derived from human studies are still lacking and the conclusions remain controversial (25). Therefore, the findings derived from streptozotocin-induced diabetes might not be readily applied to patients with diabetes mellitus if the blood-brain barrier remains intact. More in-depth studies are required to explore the possible effect of vildagliptin on the risk of dementia in humans. Second, patients with type 2 diabetes mellitus in East Asia are characterized by more remarkable β‐cell dysfunction and less insulin resistance than in Caucasians, and DPP4 inhibitors seem to exert better glycemic control in East Asians (26). In Japan, DPP4 inhibitors have become the first-line antidiabetic drugs and more than 70% of the diabetes patients are being treated with incretin-based therapies (26). Although major clinical trials suggested a neutral cardiovascular effect of DPP4 inhibitors (27) and the present study did not favor a beneficial effect of vildagliptin on dementia, DPP4 inhibitors can at least be safely used for glycemic control in older patients because of a high tolerability and a lack of hypoglycemic risk (28). Third, recent studies suggested that DPP4 inhibitors (especially vildagliptin and linagliptin) are associated with a higher risk of bullous pemphigoid (29–31). According to an observational study conducted in Taiwan, the risk factors of bullous pemphigoid in patients with type 2 diabetes mellitus seemed to be associated with using DPP4 inhibitors, having dementia and taking spironolactone (32). Therefore, DPP4 inhibitors should better be avoided in diabetes patients with dementia and/or taking spironolactone.

Some potential biases commonly seen in pharmacoepidemiological studies such as selection bias, prevalent user bias, immortal time bias and confounding by indication have been addressed in the present study. Selection bias would not be a problem because of the use of the nationwide database that covers more than 99.9% of the population. Prevalent user bias was avoided by including patients with new-onset type 2 diabetes mellitus and new users of vildagliptin (**Figure 1**).

Immortal time refers to the follow-up period when the outcome cannot happen. When the treatment status or follow-up time is inappropriately assigned, immortal time bias can be introduced (33). We tried to exclude patients with ambiguous diagnosis of diabetes mellitus by enrolling only patients who had been prescribed antidiabetic drugs for 3 or more times within one year (**Figure 1**). In the universal healthcare system in Taiwan, the information of all prescriptions in the NHI was complete during the whole follow-up period and misclassification of treatment status was not likely. Therefore, inappropriate assignment of treatment status was unlikely in the present study.

To avoid the inappropriate assignment of follow-up time, we first enrolled only patients who had been treated with antidiabetic drugs and they were followed up only after a certain period of antidiabetic treatment (**Figure 1**). This avoided the immortal time between diabetes diagnosis and the start of the use of antidiabetic drugs (i.e., a certain period when the patients could have been put on diet control or exercise and antidiabetic drugs were not used). We then excluded patients with a short follow-up duration of <6 months (**Figure 1**) to avoid the enrollment of patients with such an immortal time in the calculation of person-years. It should be pointed out that the immortal time during the waiting period between drug prescription and dispense at hospital discharge as described by Lévesque et al. (33) would not happen in Taiwan because all discharge drugs can be obtained at the hospital when the patient is discharged.

To examine whether the results might be affected by potential confounding by indication, we compared the findings between the unmatched cohort and the matched cohort based on propensity score and between the unadjusted and multivariate-adjusted models (**Table 2**). The findings seemed to be very consistent in different analyses. Analyses in subgroups of patients categorized by the tertiles of exposure parameters and by treating these parameters as continuous variables (**Table 2**) also supported a lack of association between vildagliptin and dementia. The indications and recommendations for the use of antidiabetic drugs for the treatment of type 2 diabetes mellitus have evolved over the past decades following the introduction of newer classes of antidiabetic drugs and according to the results of novel clinical trials. The ever-changing recommendations for the indications and uses of different classes of antidiabetic drugs would not confound the finding of a neutral effect of vildagliptin on dementia risk while patients enrolled during three different periods of time were analyzed separately (**Table 3**).

The present study has some other merits. First, the findings can be readily generalized to the whole population because the NHI database covers >99.9% of the Taiwan’s population. Second, self-reporting bias and recall bias could be avoided by using the medical records. Third, although detection bias because of different socioeconomic status could be a severe problem in some countries, this would not be the case in our study because the drug cost-sharing is low and can always be waived in patients with low-income, in veterans and when the patients receive prescription refills for chronic disease in our NHI healthcare system.

There are several limitations in the present study. First, we could only use the ICD-9-CM codes for disease diagnoses and no additional support from laboratory examinations was available in the database. The accuracy of the diagnosis of dementia was not known. If the misdiagnosis was non-differential between ever users and never users of vildagliptin, the estimated effect would be expected to bias towards to null (34) and a true positive or negative effect could not be shown. Therefore, the findings of the present study should better be considered as preliminary and future studies with well-verified cases are required to confirm our findings of a null effect. Second, we did not have measured data of some confounders like blood levels of glucose and insulin, fluctuation of blood glucose, indicators of insulin resistance and β-cell function, anthropometric factors, dietary pattern, nutritional status, lifestyle, smoking, alcohol drinking, family history and genetic parameters. It is recognized that the application of propensity score matching in a retrospective cohort study can never adjust for unmeasured confounders as a randomized control trial can do (35). It is impossible for the present study to assess whether the impact of unmeasured confounders could be substantial and the estimates could be misleading. Therefore, if possible, the findings of the present study should better be confirmed by a randomized control trial in the future. Third, we were not able to discern the two major types of dementia, i.e., vascular or degenerative type, because of lack of sufficient laboratory data. If the effects of vildagliptin were not the same for these two types of dementia, the estimates would be misleading by including different types of dementia. Additionally, to our knowledge, although the accuracy of diabetes diagnosis and most other comorbidities in the NHI database has been validated in previous studies (36, 37), the accuracy of dementia diagnosis remains to be validated. It would be a good future research topic to validate the related diagnostic codes of dementia in the database. Fourth, the mean age of the patients was around 58-59 years old at the start of follow-up (**Table 1**). The incidence of dementia in these patients might not be high enough to have sufficient power to detect a significant difference. It would be better to include a cohort of older age for additional study in the future. Fifth, the follow-up duration might be too short and therefore the findings should be confirmed by studies with longer follow-up duration. Finally, because the study excluded users of other incretin-based therapies, whether the findings can be applied to other DPP4 inhibitors or to glucagon like peptide-1 receptor agonists is not known.

In conclusion, the present study finds a neutral effect of vildagliptin on the association with dementia risk in Taiwanese patients with type 2 diabetes mellitus. More studies are warranted to clarify the neuroprotective effects of vildagliptin or other DPP4 inhibitors observed in *in vitro* or animal studies.

## Data Availability Statement

The datasets presented in this article are not readily available because public availability of the dataset is restricted by local regulations to protect privacy. Requests to access the datasets should be directed to C-HT, ccktsh@ms6.hinet.net.

## Ethics Statement

The studies involving human participants were reviewed and approved by The Research Ethics Committee C of the National Taiwan University Hospital (NTUH-REC No. 201805002WC). Written informed consent for participation was not required for this study in accordance with the national legislation and the institutional requirements.

## Author Contributions 

The author confirms being the sole contributor of this work and has approved it for publication.

## Funding

The study was supported partly by the Ministry of Science and Technology (MOST 107-2221-E-002-129-MY3) of Taiwan and by Novartis Taiwan. The funders had no role in study design, data collection and analysis, decision to publish, or preparation of the manuscript.

## Conflict of Interest

The author declares that the research was conducted in the absence of any commercial or financial relationships that could be construed as a potential conflict of interest.
